# Etiology of Total Knee Arthroplasty Revisions: A Two-Decade Institutional Perspective

**DOI:** 10.7759/cureus.55263

**Published:** 2024-02-29

**Authors:** Serban Dragosloveanu, Mihnea-Alexandru Petre, Bogdan Cretu, Alexandra Ana Mihailescu, Romica Cergan, Cristian Scheau

**Affiliations:** 1 Department of Orthopaedics and Traumatology, The “Carol Davila” University of Medicine and Pharmacy, Bucharest, ROU; 2 Department of Orthopaedics, "Foisor" Clinical Hospital of Orthopaedics, Traumatology, and Osteoarticular Tuberculosis, Bucharest, ROU; 3 Department of Orthopaedics, Bucharest Emergency University Hospital, Bucharest, ROU; 4 Department of Anesthesiology and Critical Care, "Foisor" Clinical Hospital of Orthopaedics, Traumatology, and Osteoarticular Tuberculosis, Bucharest, ROU; 5 Department of Anatomy, The "Carol Davila" University of Medicine and Pharmacy, Bucharest, ROU; 6 Department of Radiology and Medical Imaging, "Foisor" Clinical Hospital of Orthopaedics, Traumatology, and Osteoarticular Tuberculosis, Bucharest, ROU; 7 Department of Physiology, The "Carol Davila" University of Medicine and Pharmacy, Bucharest, ROU

**Keywords:** paraclinical investigation, pathophysiology, aseptic loosening, periprosthetic joint infection, revision surgery, total knee arthroplasty

## Abstract

Total knee arthroplasty (TKA) implant survival time is determined by various patient and implant-related factors and varies significantly in recent worldwide reports. In our study, we have included 247 TKA revisions in 203 patients performed in our hospital over the last 20 years. Multiple etiologies of revisions were identified and classified into 10 categories. Time to failure was analyzed with regard to etiology, patient demographics, and other relevant data. The overall average time to revision was 44.08 months (95% confidence interval (CI) between 33.34 and 49.82 months). Age at primary implant was negatively correlated with time to revision (hazard ratio (HR) = 1.0521 and 95% CI of HR = 1.0359 to 1.0685) and female patients showed a 1.59 times higher risk of implant failure than males. Periprosthetic joint infection was the cause of 46.56% (n=115) of revisions (out of which 12.55% (n=31) were early infections, diagnosed within the first three months), while aseptic loosening was found in 31.98% (n=79) of cases. Infection correlated with a shorter time to revision compared to aseptic loosening (p<0.05). These findings emphasize the need to intensify efforts to deliver the best patient care, select the best antibiotic regimen, and improve surgical techniques to decrease the incidence of infectious complications.

## Introduction

Total knee arthroplasty (TKA) aims at restoring the physiological weight-bearing components of the knee joint, leading to pain relief and an increase in functionality and range of motion (ROM) for individuals with knee osteoarthritis (OA) [1-3]. Knee OA is increasingly common among the general population across all continents [4-6], with a prevalence between 2.1-10.1% for men and 1.6-14.9% for women [7], and it is estimated that around 650 million individuals older than 40 years will suffer from knee OA in 2020 worldwide [7].

Given the growing number of patients undergoing knee arthroplasty for OA, the incidence of knee revision surgeries has also risen [8-11], putting a significant strain on healthcare institutions and affecting the overall quality of life for patients who underwent primary TKA [12-14]. Up to 20% of patients with primary TKA may require a revision TKA (rTKA) procedure within 10 years for a variety of reasons, ranging from infection, instability, component misalignment, polyethylene or metal wear, cement loosening, periprosthetic fracture, instability, or knee stiffness [15,16]. Among the various etiologies for rTKA, periprosthetic joint infection (PJI) stands out as one of the most prevalent and impactful on patient morbidity and mortality; however, revision prostheses have been applied to a variety of conditions [17-19]. Moreover, periprosthetic fractures can also be challenging to address due to frequent comorbidities and bone quality [20,21]. Some host factors are associated with a greater risk for early failure of primary TKAs through aseptic loosening, especially the male sex and the level of activity, while the type of fixation may increase the chance of failure in specific demographics [22,23]. This underlines the importance of studying the underlying etiologies causing the recent surge of rTKA procedures performed globally. Moreover, there are conflicting reports regarding the rankings and frequencies of rTKA causes in various areas of the world, and shifts in the indications for revisions have also been recorded [15,24-26].

There is a paucity of available literature data regarding primary TKA failure and rTKA etiologies in our country, lacking necessary information on the causes, patient-related factors, such as age, body mass index (BMI), sex, comorbidities, and the type of rTKA. Also, particular drawbacks of our national health system, such as limited access to primary care in some areas and inadequate outpatient services, can influence the prevalence of specific etiologies, such as infections [27,28]. Furthermore, a thorough comparison with reported data from other centers might reveal possible areas of improvement in our healthcare system and in the approach to primary TKA failure. Therefore, a thorough analysis of TKA failures and causes of revision may provide invaluable information regarding the profile of our population and may be relevant to similarly developed countries worldwide. These insights can come to the aid of orthopedic surgeons in supporting an informed decision regarding the potential risk factors for rTKA etiologies, which have been underexplored in the current literature.

We developed and ran an extensive study in our institution covering a period of over two decades with the primary objective of revealing the etiology of rTKA. Additionally, we studied the factors associated with significant differences in the time-to-revision.

## Materials and methods

We performed a retrospective analysis of the rTKA performed in our institution within the time period between January 2003 and April 2023. The patients were selected using our hospital electronic records, and we included all patients who underwent rTKA in our hospital, regardless of the medical center where the primary prosthetic was placed. Patients with tumor prostheses or hinged-type primary implants were excluded. We have studied several variables for each patient, including patient demographics, etiology for primary TKA, type and laterality of primary implant, dates of primary and rTKA (further expressed as time to failure), etiology and type of revision, and number of revisions for each patient.

All rTKAs were performed in our hospital between 2003 and 2023 by senior orthopedic surgeons following our institutional guidelines and national and international good practices guides. Implant information and patient clinical data were submitted to paper and electronic records. Within the specified period, we have identified a total of 261 cases of rTKA and excluded 14 cases due to incomplete data, where key demographic information or reasons for revision were not specified. The remaining patients were classified by etiology for revision into 10 categories that covered all revision causes for the study lot, as follows: early infection, chronic infection, aseptic loosening, periprosthetic fracture, ligament instability, implant wear, ROM limitation, prosthetic dislocation, pain without signs of loosening, and broken implant. In our study, PJI was diagnosed according to the Musculoskeletal Infection Society and the Infectious Diseases Society criteria for PJI secondary to TKA [29,30]. To further analyze the infection category, we have defined two entities: early infection, when it was isolated within the first three months after the initial surgery, and chronic infection, when patients reported it later than three months after the primary implant [31]. Loosening was considered when either of the femoral, tibial, or patellar components showed signs of decementation in the absence of infection, either on preoperative radiographs or documented during surgery [32,33]. We have defined post-TKA ROM limitation as flexion less than 90º, coupled with a decrease in extension by more than 10º [34].

All the data was collected from hospital records. Written informed consent was obtained from all admitted patients. The study followed the principles of medical research ethics as described in the Declaration of Helsinki in 1964, including its later amendments. All patients received the best possible medical care in relation to their disease course, complications, and comorbidities. The study was approved by the Institutional Ethics Committee of the “Foisor” Clinical Hospital of Orthopedics, Traumatology, and Osteoarticular Tuberculosis (registration no. 4976/22.05.2023).

We used descriptive statistical methods such as mean (± standard deviation) or median (range of values) when appropriate. A one-way ANOVA was used to determine differences in continuous values such as implant time to failure or patient age between subgroups of etiologies. We used the T-test or Welch test for the comparison of means between subgroups after testing for equal variances with the F-test. Pearson’s correlation test was used to assess the strength of the correlation between continuous variables. We used the Chi-square test for independence to determine whether any significant association or relationship between the variables and the study years exists. Kaplan-Meier survival analysis was used to compare the time to revision between selected patient subplots. We considered p-values equal to or less than 0.05 to be statistically significant.

## Results

**Figure 1:**
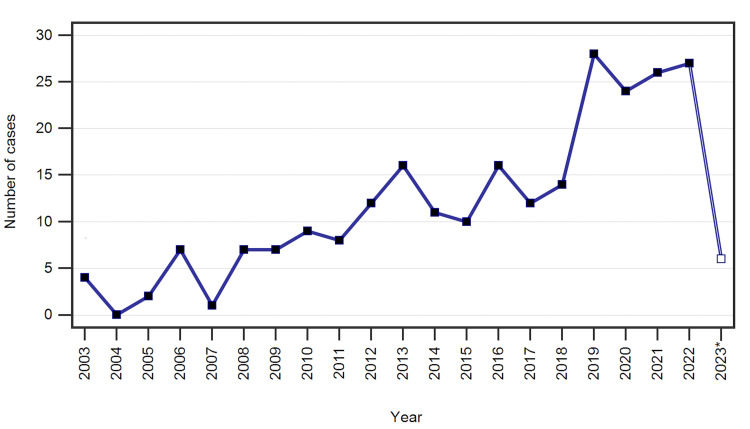
Number of rTKA cases per year throughout the study period * Only the first four months of the year 2023 were included in our study rTKA: revision total knee arthroplasty

The study group consisted of 247 revisions performed on 203 patients between January 2003 and April 2023 for primary implants placed between June 2001 and December 2022 (Figure 1). Out of the total number of primary implants, 155 were placed in our hospital. An overview of patient demographics is presented in Table 1.

**Table 1:** Patient demographics M: male; F: female; SD: standard deviation; BMI: body mass index; TKA: total knee arthroplasty

Parameter	Value
Total patients (M, F)	203 (52, 151)
Age in years at primary implant (SD)	64.15 (± 8.75)
Age in years at revision (SD)	67.81 (± 8.02)
BMI at revision	29.89 (± 5.55)
Etiology of primary TKA	
Monocompartmental arthritis (%)	13 (5.26%)
Bi- or tricompartmental arthritis (%)	167 (67.61%)
Femuropatellar arthritis (%)	30 (12.15%)
Posttraumatic arthritis (%)	19 (7.69%)
Rheumatoid arthritis (%)	6 (2.43%)
Other causes (%)	12 (4.86%)
Type of primary prosthetic	
Single condylar	7
Bicondylar	230
Patellofemoral	10
Number of revisions	
1	200 (80.97%)
2	39 (15.79%)
3	7 (2.83%)
4	1 (0.40%)

We carried out a Cox proportional-hazards regression to confirm and further analyze the influence of sex and age on implant time to revision and obtained the following results: age at primary implant had a regression coefficient (b) = 0.05, standard error = 0.01, Wald Chi-square statistic = 40.71, p<0.0001, HR = 1.05, and 95% CI of HR = 1.04 to 1.07. The HR for male patients was 0.63 (95% CI of 0.47 to 0.84); therefore, female patients had a hazard rate of implant failure approximately 1.59 times higher than males.

Laterality was not a statistically significant factor for predicting implant survival time (p=0.09). Single condylar prosthetics had an implant survival time (108 ± 59.93 months) significantly longer than bicondylar (41.91 ± 44.61 months) or patellofemoral (49.20 ± 30.97 months) prosthetics (p<0.05).

Further, we analyzed implant time to failure and patient age, BMI, and sex for each etiology for revision identified within the study period. In our patient group, we found no correlation between patient age and the reason for revision. The results are presented in Table 2.

**Table 2:** Categories of the rTKA etiology in the study group †: time to revision is depicted as median (range); age at primary implant and BMI are presented as mean ± standard deviation; ROM: range of motion; BMI: body mass index; rTKA: revision total knee arthroplasty; M: male; F: female

Reason for revision	Number of cases (%)	Time to revision (months) †	Age at primary implant (years)	BMI (kg/m^2^)	Sex (M/F)
Infection					
Early infection	31 (12.55%)	1 (0, 3)	65.77 ± 8.09	29.45 ± 5.29	15/16
Chronic infection	84 (34.01%)	18 (3, 146)	65.67 ± 8.29	30.13 ± 5.74	27/57
Aseptic loosening	79 (31.98%)	68 (3, 189)	61.41 ± 8.89	29.86 ± 5.80	14/65
Periprosthetic fracture	11 (4.45%)	24 (0, 147)	63.09 ± 10.88	29.53 ± 4.47	11/0
Ligament instability	10 (4.05%)	29.5 (0, 165)	66.50 ± 9.65	29.69 ± 5.89	2/8
Implant wear	9 (3.64%)	63 (10, 161)	66.00 ± 7.78	30.12 ± 6.21	2/7
ROM limitation	8 (3.24%)	28 (16, 98)	61.63 ± 4.41	32.44 ± 6.94	1/7
Prosthetic dislocation	7 (2.83%)	28 (1, 56)	68.43 ± 9.62	28.80 ± 4.33	2/5
Pain without signs of loosening	6 (2.43%)	29 (8, 84)	68.17 ± 7.88	28.97 ± 3.12	1/5
Broken implant	2 (0.81%)	102.5 (80, 125)	53.00 ± 9.90	26.30 ± 2.40	1/1

**Figure 2:**
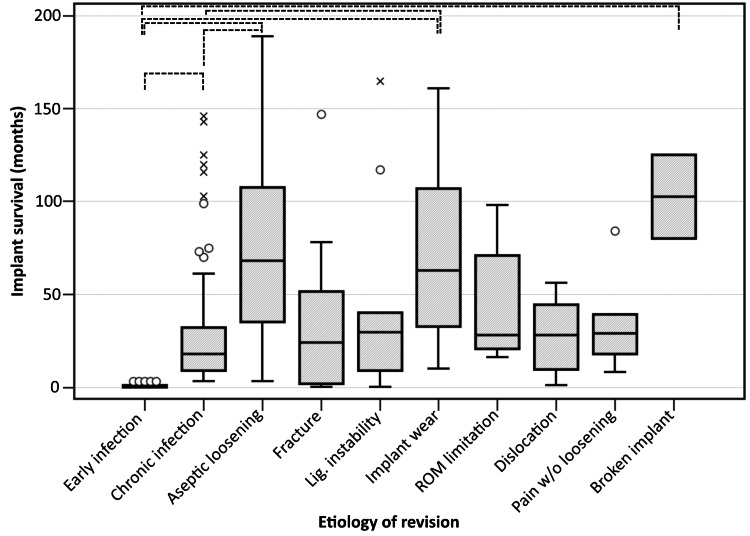
Box-and-whisker chart featuring implant survival in months, stratified according to the reason for revision Moderate (circles) and extreme (crosses) outliers are represented. Dotted lines mark statistically significant differences between pairs of categories (p<0.05)

We evaluated the implant time to revision concerning the etiology and found that, alongside early infection, chronic infection is also a reason for a shorter time to revision compared to aseptic loosening or implant wear (p<0.05) (Table 2, Figure 2).

**Figure 3:**
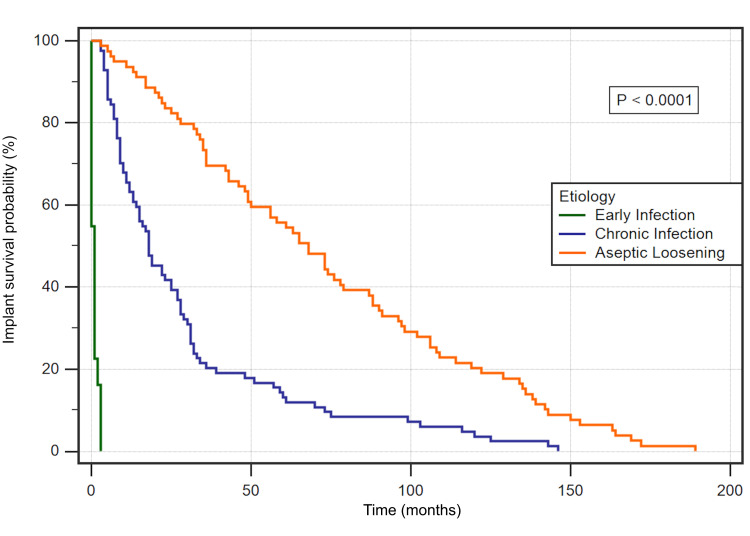
Kaplan-Meier survival curve of the time to revision for the most common etiologies of implant revision, infection and aseptic loosening Per definition, early infection cases occur within the first three months. Note the steeper curve of chronic infections where close to 80% of implants were revised during the first 36 months. In comparison, the probability of revising an implant due to aseptic loosening appears in linear correlation to the time after the primary implant. The logrank test shows Chi-square values of 235.38 and a p-value of <0.0001

A survival analysis of the time to revision was carried out among the main causes, infection, and aseptic loosening, and the results are presented in Figure 3.

**Figure 4:**
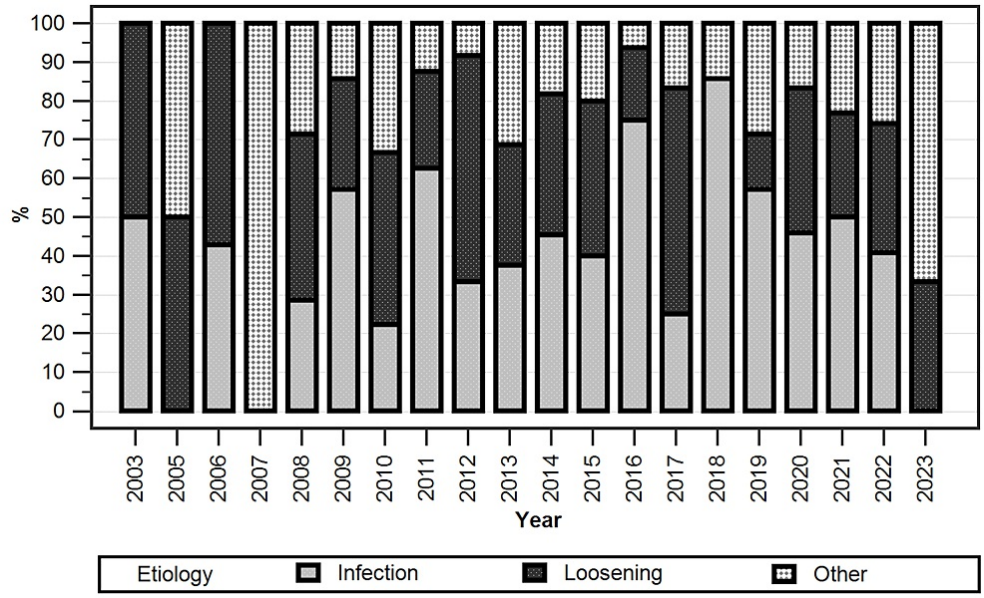
Evolution of the revision TKA etiologies throughout the study presented as stacked columns

Furthermore, a longitudinal analysis showed that, for these main three causes, there was no statistically significant change throughout the study period (p=0.08). A detailed view is presented in Figure 4, where infection, loosening, and all other causes consolidated are depicted for the duration of the study.

Since 47 cases underwent more than one revision in the studied period, we analyzed the etiology for repeated revisions and found that two cases were of early infection, 31 showed chronic infection, and seven cases were aseptic loosening, with the remainder of seven shared between other causes. None of the causes of repeated revisions were associated with a significantly different implant survival time (p=0.07). Out of the 31 cases of chronic infection as the cause of repeated revisions, eight had early infection, while the other 23 had chronic infection as the reason for undergoing the first revision.

## Discussion

TKA is considered an efficient, safe, and successful surgical technique for treating advanced osteoarthritis of the knee [35], with exceptional functional outcomes and long-term survivorship of the primary knee implant [36-39]. Understanding the etiology of TKA failure and developing ways of treatment in order to decrease the occurrence of complications that lead to rTKAs are imperative for a satisfactory quality of life for the patient post-TKA surgery.

In this study, we analyzed (1) the main etiology of primary TKA, (2) several characteristics of the primary implant, such as type and laterality, life expectancy of the implant, and type of fixation, (3) patient-related factors, such as BMI, age, and sex, (4) the number of revisions performed in the life-span of the patients, and (5) the etiology of rTKA.

In our study, the causes for rTKA were represented by early and chronic PJI, with rates of 12.55% and 34.01%, respectively, followed by aseptic loosening in 31.98% of cases. Various causes for rTKA have been described in the literature, such as polyethylene wear, ligament instability, aseptic loosening, infection, implant malalignment, periprosthetic fracture, implant wear, and ROM limitation [15,16,40]. Nevertheless, the most frequent etiologies of rTKA vary significantly between reports. Sharkey et al. [40] found polyethylene wear, aseptic loosening, and instability as the most common causes, while other studies reported the dominant mechanism of failure being PJI, occurring in 25.2% to 53% [38,41-44].

We found no correlation between patient age at primary TKA and the incidence of early or chronic PJI compared to other causes for revision. The high percentage of revisions for PJIs can be especially alarming since the burden on the public healthcare system for treating PJIs is substantial [12,45-47], and the cost of rTKA associated with PJI is four times higher than aseptic single-stage revision costs [48]. The high morbidity and mortality rates associated with PJI are a growing concern among the orthopedics community. As previously mentioned, a variety of factors can contribute to high infection rates in patients undergoing primary TKA. A recent study showed that patients receiving intra-articular injections with corticosteroid compounds in the months prior to the primary TKA have higher chances of developing PJIs [49]. Therefore, accurate recording of patient history can play a major role in the management of these patients.

The criteria used to establish PJI as the main cause in patients undergoing rTKA are also relevant in defining the infection rates in this population of patients. A recent study showed that more than 8% of patients undergoing aseptic rTKA had positive intraoperative cultures [50]. This finding shows that the ratio of infection in implant failures may be higher than considered in many studies and, at the same time, represents a challenge for the orthopedic surgeon in the management of these cases, as the decision to treat these patients or not may influence the rates of revision and implant survival [50]. Moreover, new and advanced alternative methods for diagnosing infection can help correctly classify the reason for revision as PJI and not aseptic loosening; therefore, a shift in the results of recent studies subsequently reporting higher rates of PJI may be expected [51]. Although analyzing the type of isolated organism and their resistance profile was not within the scope of this study, the most common germs recorded in our practice in PJIs consist of *Staphylococcus aureus*, both methicillin-resistant and methicillin-sensitive, and Gram-negative bacteria such as *Escherichia coli*, *Pseudomonas aeruginosa*, and *Klebsiella pneumoniae*. This distribution falls in line with literature data that reported the *Staphylococcus* genus was the most prevalent germ in more than half of total cases, with Gram-negative bacteria comprising most of the remaining cases [52,53]. Some comorbidities may influence the microorganism profile and therefore should be accounted for when analyzing infection etiology [54].

Most commonly, cases of aseptic loosening, periprosthetic fracture, ligament instability, and other “mechanical” complications after primary TKA are addressed by the surgeon who conducted the primary arthroplasty, while some advanced or complicated PJI cases are referred to our hospital regardless of the medical center of the primary TKA, due to our hospital’s higher capacity to deal with complicated cases, specialized personnel, and higher expertise in infections and complex cases. However, most likely, a bidirectional shift of patients occurred since a number of primary TKAs that require revisions for non-infectious causes were performed in other centers. It is clear that revisions for septic primary TKAs present a higher risk of complications, higher rates of mortality, and a higher risk of failure [55-57]. As such, out of 247 revisions performed in our hospital, only 155 primary TKAs were carried out by our colleagues, while the rest were performed elsewhere.

In our study, aseptic loosening of the primary TKA represented 31.98% of total revision etiologies, with a mean patient’s age at the primary TKA of 61.41 ± 8.89 years. The mean time to revision was 68 (3, 189) months, concluding that aseptic loosening was the main cause of revision for late rTKA, while chronic infection was the prime etiology for early revision, with an onset at 18 (3, 146) months. However, we found no correlation between obesity (BMI ≥ 30 kg/m^2^) and an increased risk of aseptic loosening, although a consensus on this topic has not yet been reached [22,58].

The periprosthetic fracture occurred in just 4.45% of total rTKA cases, with a median time to revision of 24 (0, 147) months. One patient suffered a traumatism falling from the same height on the second day postoperatively, resulting in a left femur supracondylar fracture.

Among the 247 revision cases assessed, 10 cases of ligament instability required revision surgery, accounting for approximately 4% of cases. Patients experiencing this complication after TKA surgery had an average time to revision of 29.50 (0, 165) months, with one case being iatrogenic, resulting in early revision involving Legacy Constrained Condylar Knee prostheses.

The average time to revision in our study was 44.08 months, which aligns with previous literature studies [59,60]. The patients undergoing rTKA procedures were on average 67.81 (± 8.02) years old at the moment of surgery; additionally, 15.79% of patients required second revision surgery, and 2.83% of patients required a third intervention. This raises concerns, especially when considering the mean age of the patients who require rTKA surgery and the rising average life expectancy in our country [61,62]. Given the increasing number of patients living longer, healthier, and more active lives, it is becoming increasingly apparent that a large number of individuals who underwent primary TKA procedures will eventually require revision surgery for their primary implant.

An examination of the number of cases per year performed in our center revealed a growing number of revision cases. This is mainly explained by the steady increase in the operational capacity and number of admissions and surgeries performed per year during the last two decades. Our longitudinal analysis of rTKA causes showed that the percentage of PJIs tends to increase throughout the analyzed period, while loosening shows a decreasing tendency. This finding is similar to other reports and reveals that, on the one hand, advancements in implant design and manufacturing have decreased the incidence of mechanical failures while, on the other hand, infections have become the main cause for rTKA in major centers worldwide, due to a variety of factors that can be categorized as patient-related, such as rheumatoid arthritis, diabetes mellitus, peripheral vascular disease, hypertension, or congestive heart failure, or surgery-related, such as the operative time, intraoperative bleeding, local antisepsis, or choice of prophylactic antibiotic [39,63-68]. These main factors were also identified in our patient lot, either alone or combined; however, the analysis of the clinical parameters and risk factors was outside the scope of this paper. Nevertheless, while significant progress in rTKA indications, surgical planning and approach, robotic-assisted surgical options, management, and follow-up of patients has been recorded throughout the last 20 years, the definitive role and contribution of these improvements to primary TKA failure remain to be established [69-77].

Although a consensus has not been reached regarding the ideal approach for antibiotic prophylaxis in TKA [78,79], our patients receive a dual prophylactic antibiotic combination consisting of a cephalosporin, such as cefazoline, cefoxitin, or ceftriaxone, and vancomycin. Antibioprophylaxis for rTKA consists of a targeted antibiotic regime if bacteriologic etiology is documented before surgery.

In our study, the female-to-male ratio of patients was approximately 3:1, and women had an elevated risk of implant failure. These findings are consistent with other epidemiological studies citing a higher incidence of TKA in females [80-84]. However, male patients had a significantly shorter time to revision, which is consistent with reports of higher physical activity after TKA in men compared to women [85]. Nevertheless, there is a lack of consensus regarding sex-based differences in primary and rTKA, the risk factors for implant failure, and the long-term outcomes of rTKA [17,86,87].

While surgeon-handedness may play a role in the outcome of left vs. right primary TKA [88,89], there is insufficient data to suggest that either side is more frequently affected by a particular complication; our study supports the finding that laterality does not influence the time-to-revision.

One of our study limitations was that we performed a single-center analysis, where data was collected from revisions solely performed in our hospital. This approach may restrict the applicability of the findings to a broader population and may not reflect the general context of the addressed issue among other healthcare providers. However, we included a large number of rTKA procedures over a time span of 20 years in order to increase the patient population and study significance; in itself, this may represent a source of bias due to various changes in technology, surgical techniques, prophylaxis protocols, and other variables and may limit the relevance of the study to current clinical practice. Furthermore, strict protocols for collecting, recording, and archiving clinical data were followed to ensure that the relevant information used in our study was accurate. However, the retrospective approach may introduce certain limitations in the accuracy of the recorded data and could affect the validity of the results. Moreover, we did not have complete access to all clinical and imaging data in patients who underwent primary TKA in other centers in order to assess whether implant malposition or other perioperative complications were recorded and if these played a role in the time-to-revision for these patients. The exclusion of patients due to incomplete data may introduce a selection bias and might affect the overall distribution of certain etiologies identified in our study. Further research, including the analysis of implant model and material versus time-to-revision, could reveal interesting results, and a large registry-based study including recent and contemporary implants may be useful in understanding the effectiveness of the prostheses currently in use. Stratification of patient-related factors based on implant type and model could further reveal useful insight into the reasons for primary TKA failure. Collecting additional details regarding the infectious agent, antibiotic resistance, and genetic expression profiles may improve our understanding of the specific strains and the associated immune response. Multi-centric and international clinical studies may reveal more accurate data on the rTKA etiology and implicated factors and may overcome population selection biases as well as isolate and minimize the influence of other confounding variables. Several recent studies on rTKA have obtained very large patient cohorts and were able to provide an overview of the addressed issue. However, they admittedly met limitations regarding the variability of implant models and the heterogeneity of certain subgroups, which hindered the data analysis [90,91].

## Conclusions

PJI was the main etiology of rTKA in our study, identified in almost half of the cases, out of which approximately a third were early infections, occurring in the first three months after the primary TKA. The second most common cause of revision was aseptic loosening, in almost a third of all cases. Although the study population was limited to our country, these findings are in line with other reports around the world, and they underscore the critical importance of infection prevention measures in orthopedic procedures. Our results support those of larger studies that have emphasized the critical role of optimizing preoperative screening as well as the administration of prophylactic antibiotics and proper intraoperative techniques to reduce the risk of revision due to infection.

PJIs remain a significant concern in TKA for the international orthopedic community; subsequently, the horizons for new procedures, implants, and research directions are of utmost importance. Research can focus on developing a more effective antimicrobial coating for implants, antibiotic-loaded implants, preoperative screening, and risk assessment. As new technologies for implants are developed, surgical techniques and instrumentation can also evolve and contribute to the overall reduction of TKA complications, e.g., minimally invasive approaches, improved sterilization methods, and surgical theater optimization. The burden of a successful TKA also lies on the patient, emphasizing the importance of patient education, adherence to postoperative protocols, proper wound care, and early signs of infection detection. These are a few methods for the patient to play an active role in surgical procedure satisfaction.

The results of our study warrant a series of recommendations for orthopedic surgeons in order to lower the necessity for rTKA, such as adopting minimally invasive approaches, reducing surgical duration, and reaching a better understanding of bacterial infections specific to each practice or region. Moreover, screening protocols for identifying patients susceptible to infection, such as those suffering from diabetes, rheumatoid arthritis, and chronic corticosteroid therapy, are of utmost importance for a safe and satisfying surgical procedure.
